# A Multifunctional ε-Polylysine/Hyaluronic Acid Hydrogel Promotes Diabetic Wound Healing by Orchestrating Multidimensional Synergy

**DOI:** 10.3390/pharmaceutics18040473

**Published:** 2026-04-13

**Authors:** Zelong Li, Yiqin Wang, Yifan Zhou, Hongze Liang, Xianwu Chen, Xiao Wang, Ziyu Liu, Lingling Zhao

**Affiliations:** 1 School of Materials Science and Chemical Engineering, Ningbo University, Ningbo 315211, China; 2311260116@nbu.edu.cn (Z.L.); 2411260044@nbu.edu.cn (Y.W.); 2211260221@nbu.edu.cn (Y.Z.); lianghongze@nbu.edu.cn (H.L.); 2 The Affiliated Hospital of Medical School, Ningbo University, Ningbo 315211, China; fychenxianwu@nbu.edu.cn; 3 Health Science Center, Ningbo University, Ningbo 315211, China; wangxiao@nbu.edu.cn; 4 Beijing Advanced Innovation Center for Biomedical Engineering, School of Engineering Medicine, Beihang University, Beijing 100191, China

**Keywords:** hydrogel dressing, diabetic wound healing, antioxidant, anti-inflammatory, antibacterial, angiogenesis, ferroptosis

## Abstract

**Background/Objectives**: Diabetic wound healing faces significant challenges due to the harsh microenvironment of wounds such as high blood glucose levels, excessive inflammation, persistent infection, upregulated reactive oxygen species (ROS), and damaged new blood vessels. Therefore, developing hydrogel dressings with microenvironmental regulation functions has become an important strategy in treating diabetic wounds. **Methods**: In this study, an ultraviolet in situ crosslinked hydrogel (D@H/E) was developed using methacrylic anhydride modified hyaluronic acid (HA-MA) and glycidyl methacrylate modified ε-polylysine (EPL-GMA), loaded with the iron chelating agent desferrioxamine (DFO). The physicochemical and biochemical properties of the hydrogel were comprehensively characterized, and its efficacy as a dressing for diabetic wounds was evaluated in a STZ-induced hyperglycemic mouse model. **Results**: This hydrogel demonstrated remarkable multidimensional effects by alleviating oxidative stress damage, inhibiting bacterial infection, regulating inflammatory responses, mitigating ferroptosis, and promoting cell migration and tubule formation. Specifically, the DFO-loaded hydrogel achieved a high DPPH radical scavenging efficiency of 80.8% and exhibited excellent antibacterial activity, with over 99.8% inhibition against both *S. aureus* and *E. coli*. In streptozotocin (STZ)-induced diabetic mice, the hydrogel accelerated wound closure to near completion by day 14. Mechanistically, it significantly upregulated CD206 expression to promote M2 macrophage polarization, upregulated the expression of angiogenesis-related factors to promote angiogenesis at the wound site, and enhanced GPX4 expression to alleviate ferroptosis. **Conclusions**: By orchestrating multi-dimensional synergy that combines ROS scavenging, infection control, immune regulation, and anti-ferroptosis, this D@H/E hydrogel system effectively remodels the harsh diabetic wound microenvironment, offering a promising platform for chronic wound management.

## 1. Introduction

Metabolic syndrome based on diabetes has gradually become a serious chronic disease that significantly affects human health. Poor wound healing caused by diabetes is one of its main complications [1]. It is reported that there are approximately 18.6 million people worldwide affected by diabetic wounds each year, and severe cases of these wounds can lead to amputation of the lower limbs, causing significant social and economic burdens [2]. Chronic diabetic wounds can persist for several months to several years and often recur, leading to a decline in quality of life and loss of skin and mucosal functions [3]. Wound healing is the basic stage of skin injury reconstruction and is a very complex and regular process, which includes four overlapping stages: hemostasis, inflammation, proliferation, and remodeling in normal wound healing [4]. Due to the presence of hyperglycemia, diabetic wounds are often accompanied by persistent inflammatory responses, oxidative stress damage, angiogenesis disorders, and extremely high risks of bacterial infections, which result in the inhibition of dermal and epidermal cell proliferation in diabetic wounds, making the recovery and healing of diabetic wounds extremely difficult [5,6].

In recent years, a variety of treatment methods for diabetic wounds have emerged, including antibacterial therapies [7], passive wound patch [8], stem cell therapies [9], and various wound dressings [10]. Among these treatment approaches, hydrogel dressings have demonstrated significant potential due to their unique advantages in the wound healing process [11,12]. Hydrogels possess excellent biocompatibility and water retention properties, and can provide a moist environment at the wound site, absorb exudates from the tissue, allow oxygen to pass through, and act as a barrier against microbial invasion [13]. Through structural and biochemical adjustments, multifunctional hydrogels with various properties can be designed, functioning in aspects such as antioxidant, antibacterial, inflammation regulation, and angiogenesis promotion, to facilitate the repair of diabetic wounds [14].

Persistent inflammation is a hallmark of chronic non-healing wounds and is an important factor contributing to the difficulty in wound healing [15]. Macrophages play a crucial role in wound healing and immune regulation. Typically, macrophages transfer from an inflammatory phenotype (M1) to an anti-inflammatory phenotype (M2) in the late stage of wound healing [16]. M2 macrophages are particularly important for wound vascular reconstruction as they can release angiogenic-related factors and anti-inflammatory cytokines [17]. Due to the unique hyperglycemia environment of diabetes, the polarization process of macrophages from M1 phenotype to M2 phenotype is hindered [18,19]. The accumulation of M1 macrophages leads to the release of pro-inflammatory cytokines and the production of ROS, thereby triggering oxidative stress and cell damage, resulting in prolonged inflammatory periods and vascular lesions [20]. Therefore, using immunomodulatory strategies to provide an anti-inflammatory environment is an effective method for diabetic wound healing. Hyaluronic acid (HA) is a natural polysaccharide with good biocompatibility, biodegradability and non-immunogenicity, and is widely used in biomedical application [21]. HA can regulate inflammatory responses, promote cell migration and proliferation, and participate in the angiogenesis process, playing an important role in the repair of diabetic wounds [22,23,24]. The high water absorption of HA enables it to absorb exudate from the wound, maintain the wound moist, and facilitate wound healing [25]. In addition, the carboxyl and hydroxyl groups on HA are convenient for chemical modification (such as carbon-carbon double bond modification) to improve its properties and construct various functional hydrogel materials [26].

Bacterial infection is a significant cause for diabetic wounds to develop into chronic non-healing wounds [27]. Currently, antibiotics remain the main method for treating bacterial infections in diabetic wounds. However, long-term antibiotic treatment can lead to bacterial resistance, thereby exacerbating the difficulty in wound healing. Therefore, hydrogels with antibacterial properties have become a major research focus. ε-Polylysine (EPL) is a polypeptide with broad-spectrum antibacterial activity, and demonstrates significant advantages in the treatment of diabetic wounds [28]. EPL has been proven to have a rapid bactericidal effect on drug-resistant Gram-negative bacteria, Gram-positive bacteria, and fungal pathogens, including those bacterial strains classified as serious and urgent threats by the Centers for Disease Control and Prevention, which translates into greater efficacy and reduce the emergence of drug resistance [29]. Additionally, EPL has excellent biocompatibility and biodegradability since it can be degraded into harmless lysine in the body without causing significant inflammatory responses or toxicity [30]. It is reported that EPL not only effectively inhibits bacterial infections but also accelerates wound healing by promoting angiogenesis and regulating immune responses in diabetic wound repair [31]. Therefore, EPL provides an ideal and effective raw material choice for the construction of diabetic wound dressings.

**Scheme 1:**
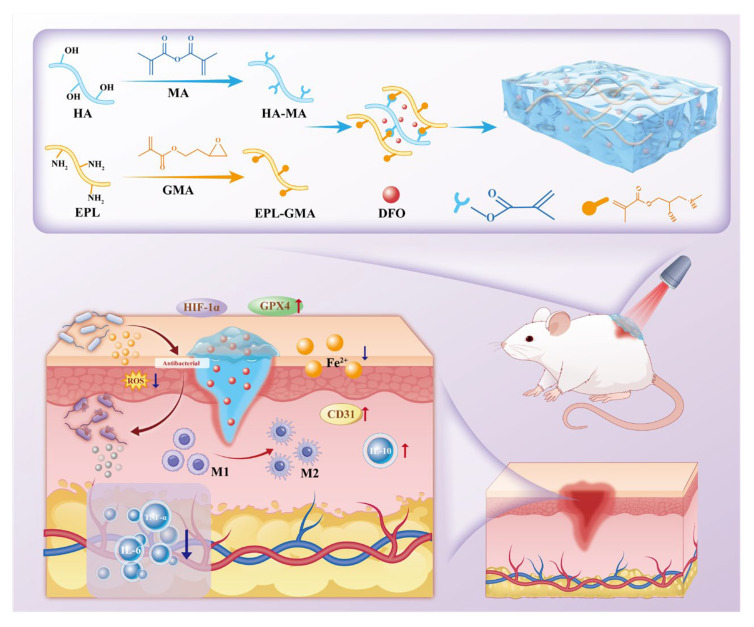
Schematic diagram of the preparation of ultraviolet in situ cross-linked multifunctional hydrogel, and the mechanism of hydrogel to promote diabetic wound healing through multi-dimensional synergistic effects.

In this study, HA and EPL are selected as the raw materials to construct the multifunctional hydrogel for the repair of diabetic wounds. Carbon-carbon double bonds were introduced into the skeleton of HA and EPL through chemical modification, and the hydrogel was prepared through in situ crosslinking triggered by UV light. DFO, an FDA approved iron chelator was encapsulated into the hydrogel to enrich its versatility. DFO can reduce the intracellular concentration of iron ion and alleviate oxidative stress damage by reducing the generation of reactive oxygen species (ROS) mediated by iron ions, effectively alleviating ferroptosis [32]. DFO can also significantly accelerate the formation of new blood vessels under both normal and pathological conditions by upregulating the expression of hypoxia-inducible factor-1α and its downstream genes [33]. It is hypothesized that combined the multiple effects of HA, EPL and DFO, this hydrogel dressing is expected to accelerate the healing of diabetic wounds through multi-dimensional synergistic effects by reducing oxidative stress, improving inflammatory responses, inhibiting bacterial infections, and promoting angiogenesis (Scheme 1). Herein, the physical, chemical and biochemical properties of the hydrogel were characterized, and its effectiveness as a diabetic wound dressing was evaluated in a STZ-induced hyperglycemic mouse model.

## 2. Materials and Methods

### 2.1. Materials

Hyaluronic acid (HA, Mw 400 kDa), epsilon-poly-L-lysine (EPL, Mw 3000 Da), and deferoxamine (DFO) were purchased from Shanghai Macklin Biochemical Technology Co., Ltd. (Shanghai, China). Methacrylic anhydride (MA) was purchased from Shanghai Aladdin Biochemical Technology Co., Ltd. (Shanghai, China). Glycidyl methacrylate (GMA) was purchased from Shanghai Yuan Ye Biotechnology Co., Ltd. (Shanghai, China). Lithium phenyl-2,4,6-trimethylbenzoylphosphinate (LAP) was purchased from Beijing InnoChem Science & Technology Co., Ltd. (Beijing, China). The Dulbecco modified Eagle’s Medium (DMEM) was obtained from Promega Corporation (Madison, WI, USA). Fetal bovine serum (FBS) was purchased from Gibco (Thermo Fisher Scientific, Waltham, MA, USA).

### 2.2. Synthesis of Methacrylic Anhydride Modified HA (HA-MA)

HA-MA was synthesized according to previous reports with modification [34]. Briefly, 2 g HA was dissolved in 100 mL deionized water under agitation and incubated in an ice/water bath. Then, 7 mL methacrylic anhydride was added dropwise under agitation. The mixture was kept agitating for 24 h, and the pH value of the solution was maintained at 8 to 9 by adding 1 M NaOH solution every hour within the first 12 h of the reaction. After another 12 h of reaction, the mixture was poured into 500 mL ethanol and kept at −20 °C overnight. The precipitation of HA-MA was collected by centrifugation (5000 r/min, 10 min). The obtained HA-MA was dissolved in deionized water, and dialyzed for 3 days in a dialysis tub (cut-off molecular weight 8000 Da), with water changes every 2 h. The residues were freeze-dried to obtain HA-MA with a yield of 87.7%, and the product was stored at 4 °C.

### 2.3. Synthesis of Glycidyl Methacrylate Modified EPL (EPL-GMA)

EPL-GMA was synthesized according to previous reports with modification [35]. Briefly, 2 g EPL was dissolved in 20 mL deionized water under agitation, and 1 mL glycidyl methacrylate was added dropwise. The mixture was reacted at 60 °C for 24 h. The residues were transformed to a dialysis tub (cut-off molecular weight 1000 Da), and dialyzed for 3 days with water changes every 2 h. The residues were freeze-dried to obtain EPL-GMA with a yield of 68.4%, and the product was stored at 4 °C.

### 2.4. Preparation of Hydrogels

HA-MA and EPL-GMA were dissolved in deionized water at appropriate concentrations, respectively. DFO and LAP were added to the EPL-GMA solution. The two solution was mixed at equal volume under agitation to form a uniform solution, and then irradiated under 365 nm ultraviolet laser for ~10 s to form hydrogel. The final concentrations of LAP and DFO in the hydrogel were 1 mg mL^−1^ and 2 mg mL^−1^, respectively. The DFO-loaded hydrogels were named D@H/E. EPL solution containing LAP but no DFO was used to prepare hydrogels without DFO, and the hydrogel was named H/E.

### 2.5. Characterization

The residual monomers in purified HA-MA and EPL-GMA were analyzed using thin-layer chromatography (TLC). HA-MA and EPL-GMA samples were extracted with acetone to isolate residual monomers. After centrifugation, the supernatant was collected for spotting, followed by development in the mobile phase and visualization under a 254 nm UV lamp. The MA standard and HA-MA extract were analyzed using a developing solvent system of n-hexane:dichloromethane = 50:50 (*v*/*v*), while the GMA standard and EPL-GMA extract were analyzed using n-hexane:dichloromethane = 60:40 (*v*/*v*).

The chemical structures of EPL-GMA and HA-MA were characterized by ^1^H NMR (Bruker AVANCE III 400 MHz, Bruker BioSpin, Ettlingen, Germany) using D_2_O as the solvent at a sample concentration of 10 mg mL^−1^. FTIR spectra were recorded using a Nicolet 6700 spectrometer (Thermo Nicolet, Madison, WI, USA). The samples were mixed with KBr at a ratio of 1:100 and pressed into transparent pellets. The FTIR measurements were performed with 32 scans in the wavenumber range of 4000 to 500 cm^−1^. The cross-sectional morphological features of the hydrogel networks were characterized by a field emission scanning electron microscope (SEM, Hitachi S4800, Hitachi High-Tech Corp., Tokyo, Japan). Prior to observation, the freeze-dried hydrogel samples were sputter-coated with a 10 nm thick layer of gold. The SEM imaging was performed at an acceleration voltage of 10 kV, a working distance of 10 mm, and a magnification of 100×. The average pore size and porosity of the hydrogels were quantified from the SEM images using ImageJ 1.54g software. For each group, ten representative pores were randomly selected for diameter measurement to calculate the mean pore size, and the porosity was determined by calculating the ratio of the total pore area to the total cross-sectional area across multiple representative SEM micrographs (*n* = 3 per group).

### 2.6. Rheological Test

The rheological behavior of the hydrogels was characterized using an HR-S rheometer (TA Instruments, New Castle, DE, USA). The H/E hydrogels with various concentrations were transferred onto a parallel-plate with a diameter of 25 mm. The storage modulus (G′) and the loss modulus (G″) of the hydrogels were measured at 37 °C. Frequency sweep tests were performed at a strain of 2% within the range of 0.1 to 100 rad/s.

### 2.7. In Vitro Drug Release Behavior

1 mL of the prepared D@H/E hydrogel with different concentrations were placed in a vial containing 10 mL of PBS solution (0.01M, pH 7.4) and were placed in a water bath at 37 °C. At the predetermined time points, 2 mL of the release solution was taken out and 2 mL of fresh PBS was added to maintain the total volume of the release medium unchanged. The amount of released DFO is measured according to previous report [36]. Briefly, the collected release solution was reacted with the same volume of 3 mM FeCl_3_ solution for 10 min. The absorbance of the DFO-Fe^2+^ complex at 432 nm was measured using a microplate reader. The content of DFO in the release medium at various time points is calculated using the linear standard curve of DFO-Fe^3+^ (Figure S1).

### 2.8. Free Radical Scavenging Test In Vitro

#### 2.8.1. DPPH Free Radical Scavenging Test

400 μL H/E and D@H/E hydrogel were incubated in 2 mL DPPH ethanol solution (0.1 mM) for 30 min at 37 °C in the dark. Then, the supernatant was taken out and the absorbance at 517 nm was measured using recorded using a microplate reader (BioTek Instruments, Winooski, VT, USA). The DPPH scavenging efficiency was calculated by Formula (1).

(1) $$DPPH\;scavenging\;efficiency=\frac{A_{D}-A_{DH}}{A_{D}}\times 100\%$$

where *A_D_* and *A_DH_* represent the absorbance of DPPH solution before and after incubated with hydrogels, respectively.

#### 2.8.2. ABTS Free Radical Scavenging Test

7 mmol ABTS solution and 2.4 mM potassium persulfate solution were mixed in equal volume at room temperature in the dark for 24 h to form a stable ABTS^+^ radical mother solution. Then, the mother solution was diluted 10 times with PBS (0.01 M, pH = 7.4) to obtain the detection working solution. 400 μL H/E and D@H/E hydrogel samples were added to 2 mL of the ABTS^+^ working solution, and incubated in the dark for 30 min. The reaction solution was transferred to a 96-well plate and the absorbance at 734 nm was recorded using a microplate reader. The ABTS radical scavenging efficiency is calculated by Formula (2).

(2) $$ABTS\;scavenging\;efficiency=\frac{A_{A}-A_{AH}}{A_{A}}\times 100\%$$

where *A_A_* and *A_AH_* represent the absorbance of ABTS solution before and after incubated with hydrogels, respectively.

### 2.9. Intracellular Antioxidant Test

Raw264.7 cells were seeded on a 24-well plate at 1 × 10^4^ cells per well. After 24 h of incubation, the medium was removed, DMEM containing 200 μM H_2_O_2_ was added and incubated for another 24 h. Then, the medium was removed, hydrogel extract solutions (400 μL hydrogel incubated in 4 mL DMEM at 37 °C for 24 h) were added and incubated for 24 h. Subsequently, the hydrogel extract solutions were removed, DCFH-DA (10 μM in serum-free DMEM) was added and incubated for 20 min. Finally, the cells were rinsed three times with PBS and observed under a fluorescence microscope (NiKon DS-Qi2, Nikon Instruments Inc., Melville, NY, USA). The excitation wavelength was 488 nm. A blank group (cells without H_2_O_2_ treatment) and a control group (cells treated with H_2_O_2_ and incubated in DMEM) were set as controls.

### 2.10. Biocompatibility Assess

#### 2.10.1. CCK-8 Assay

NIH 3T3 cells were seeded on a 96-well plate at 5 × 10^3^ cells per well and incubated in complete DMEM for 24 h. Then, the DMEM was replaced with 100 μL hydrogel extract solutions and incubated for 1, 2 and 3 days. After that, the cells were rinsed twice with PBS and incubated with DMEM containing 10% CCK-8 assay for 2 h. The absorbance of each well was recorded using a microplate reader, and the cell viability was calculated by Formula (3).

(3) $$Cell\;viability=\frac{A_{s}-A_{b}}{A_{c}-A_{b}}\times 100\%$$

where *A_s_* and *A_c_* represent the absorbance of cells incubated with hydrogel extract solutions or DMEM, respectively. *A_b_* represent the absorbance of DMEM without cells.

#### 2.10.2. Live/Dead Cells Staining

NIH 3T3 and Raw 264.7 cells were seeded on a 24-well plate at 1 × 10^4^ cells per well and incubated in complete DMEM for 24 h. Then, the DMEM was replaced with 500 μL hydrogel extract solutions and incubated for another 24 h. After that, the cells were rinsed twice with PBS and incubated with DMEM containing 2 μM Calcein-AM and 4.5 μM PI at 37 °C in the dark for 20 min, and then observed using a fluorescence microscope. The excitation wavelength was 490 and 545 nm for Calcein-AM and PI, respectively.

#### 2.10.3. In Vitro Hemolysis Test

Fresh mouse blood was centrifuged at 1000 rmp/min for 10 min to obtain red blood cells. The red blood cells were then washed three times with PBS and diluted to a final concentration of 5% (*v*/*v*). Subsequently, 0.2 mL of hydrogel samples were put in a 24-well plate, and 2 mL of red blood cell suspension was added to each well. After incubation for 2 h, the red blood cell suspension was centrifuged at 1000 rmp/min for 10 min. The supernatant was then collected and the OD value at 540 nm was measured. The red blood cells treated with PBS were used as the negative control group, and the red blood cells treated with 0.1% Triton X-100 (YuanYe Bio, Shanghai, China) were used as the positive control group. The hemolysis rate was calculated by Formula (4):

(4) $$Hemolysis\;rate=\frac{A_{H}-A_{B}}{A_{T}-A_{B}}\times 100\%$$

where *A_H_, A_B_* and *A_T_* represent the OD value of red blood cells incubated with hydrogels, Triton X-100 and PBS, respectively.

### 2.11. Evaluation of M2 Macrophage Polarization

Raw 264.7 cells were seeded on a 24-well plate at 1 × 10^4^ cells per well and incubated in complete DMEM for 12 h. Then, the DMEM was replaced with LPS solution (1 μg mL^−1^ in serum-free medium) and incubated for 24 h. Subsequently, the LPS solution was removed and hydrogel extract solutions was added. After incubation for another 24 h, the cells were incubated with CD86 and CD206 antibodies, and then observed using a fluorescence microscope or detected using a flow cytometer (Beckman Coulter, Brea, CA, USA) according to the standard procedure.

### 2.12. Anti-Bacterial Test

The antibacterial activity of the hydrogel against Gram-positive *Staphylococcus aureus* (*S. aureus*) and Gram-negative *Escherichia coli* (*E. coli*) was evaluated using the plate colony counting method. In brief, the sterilized hydrogel was placed in a 24-well plate, followed by the addition of 30 μL of bacterial suspension (5 × 10^6^ CFU mL^−1^) containing the culture medium. The mixture was cultured at 37 °C for 3 h. Then, 100 μL of the diluted bacterial solution was evenly spread on the LB solid culture medium and cultured at 37 °C for 24 h. The colonies on the solid culture medium were photographed and counted. The bacterial suspension treated with PBS was used as the control. The antibacterial ratio was calculated by Formula (5).

(5) $$\mathrm{Antibacterial}\;\mathrm{ratio}=(1-\frac{N_{h}}{N_{p}})\times 100\%$$

where *N_h_* and *N_p_* represent the number of colonies treated with hydrogel and PBS, respectively.

### 2.13. Cell Migration Test

#### 2.13.1. Scratch Assay

NIH 3T3 cells were seeded on a 24-well plate at 1 × 10^5^ cells per well and cultured in DMEM for 24 h. A horizontal scratch was created using a sterile 10 μL pipette tip. Each well was gently washed with PBS to remove the cell debris, and then cultured with different hydrogel extract solutions. The cell migration status at different time points was observed and photographed using a microscope, and the cell migration area was calculated.

#### 2.13.2. Transwell Assay

The NIH 3T3 cells were starved for 24 h, then seeded in the upper chamber at 2 × 10^4^ cells per well and cultured in serum-free medium. The lower chamber was supplemented with different gel extract solutions to induce cell migration. After incubation for 24 h, the cells in the lower chambers were rinsed with PBS three rinses, fixed in 4% paraformaldehyde for 10 min, stained with 0.1% crystal violet for 10 min, and rinsed with PBS until the excess crystal violet was removed. Finally, the cells were observed and photographed.

### 2.14. Tubular Formation Test

30 μL of matrigel was added to a 24-well plate and incubated at 37 °C to form a gel. HUVEC were seeded on the above gel at 2 × 10^4^ cells per well, and 400 μL of hydrogel extract solutions were added. After incubation for 12 h, the cells were observed using a microscope. The total length of the tubes, the number of tubes formed, and the number of tube nodes were analyzed using ImageJ software.

### 2.15. Mitochondrial Membrane Potential Test by JC-1 Staining

Raw 264.7 cells were seeded on a 24-well plate and incubated at 1 × 10^4^ cells per well and incubated for 24 h. Then, the medium was removed and replaced with DMEM containing 200 μM H_2_O_2_. After incubation for 24 h, the medium was removed and replaced with different hydrogel extract solutions. After another 24 h of incubation, the hydrogel extract solution was removed and the cells were incubated with JC-1 staining working solution for 20 min. Finally, the cells were washed with JC-1 staining buffer and observed using a fluorescence microscope. A blank group (without H_2_O_2_ treatment) and a control group (cells treated with H_2_O_2_ and incubated in normal medium) were set as controls.

### 2.16. Evaluation of Wound Healing in Diabetic Mouse

Diabetic wound model was established using BALB/c mice (female, 6–8 weeks, 20–25 g) according to our previous work [37]. All animal experiments were approved by the Animal Ethics Committee of Ningbo University (Certificate number: NBU20240365). The diabetic wound mice were randomly divided into three groups (*n* = 5) and received different treatments. In the control group, wounds were treated with 3M commercial film dressing; in the H/E group, wounds were treated with H/E hydrogels; in the D@H/E group, wounds were treated with D@H/E hydrogel. The 3M dressing was directly applied to the wound surface. For the hydrogels dressing, the mixed polymer solution was dripped onto the wound surface to cover the entire wound area, and then exposed under a UV light for 10 s to form a hydrogel. Each group received a single treatment of the dressing, with no subsequent replacements. Daily photos of the mice’s wounds were taken, and the wound area was measured using ImageJ software.

### 2.17. Histopathological, Immunofluorescence and Immunohistochemical Analysis

On the 7th and 14th days after treatment, the surrounding tissues of the wound sites in each group were collected, fixed in 10% formalin solution, dehydrated with a gradient of alcohol, and then embedded in paraffin. The samples were sectioned into 5 μm thick sections, stained with hematoxylin-eosin (H&E) and Masson’s trichrome (MT) for histopathological analysis. The expression levels of Ki67, HIF-1α, VEGF, CD31, M2-type macrophage markers (CD206) and M1-type macrophage markers (CD86) at the regenerative tissues were observed and quantitatively analyzed.

### 2.18. Western Blotting Analysis of GPX4 in Regenerated Tissues

On the 7th day after treatment, the surrounding tissues of the wound sites in each group were collected, the subcutaneous fat and connective tissue were quickly removed. The tissues were washed with pre-cooled PBS, and homogenized at −20 °C in RIPA lysis buffer containing protease inhibitors to extract total proteins. Equal amounts of protein were loaded to the SDS-PAGE electrophoresis using 10% separating gel. The electrophoresis conditions were 80 V for 30 min, then 120 V for 50 min. The membrane was spun at 320 mA for 50 min on a wet basis. The primary antibody was incubated at 4 °C overnight, the membrane was blocked with milk for 2 h, and the secondary antibody was incubated at room temperature for 1 h. Finally, the results were developed using a chemiluminescence system and corrected with the internal reference protein (β-Actin).

### 2.19. Statistical Analysis

The data are presented as mean ± standard deviation (*n* ≥ 3). One-way analysis of variance (ANOVA) was performed using GraphPad 10.0, followed by Tukey’s test. Statistical significance was set as * *p* < 0.05, ** *p* < 0.01, and *** *p* < 0.001.

## 3. Results and Discussion

### 3.1. Synthesis and Characterization of HA-MA and EPL-GMA

HA-MA was synthesized through the esterification of HA and MA, and EPL-GMA was synthesized through the acylation of EPL and GMA (Figure S2), and the samples were purified by dialysis according to previous reports [34,35]. To assess the purity of the polymers, residual monomers in purified HA-MA and EPL-GMA were analyzed using TLC. HA-MA and EPL-GMA samples were extracted with acetone to isolate residual monomers, taking advantage of the good solubility of MA and GMA in acetone, while the macromolecular remained insoluble. As shown in Figure S3a, the MA standard exhibited a clear spot under UV light, whereas no corresponding spot was detected in the HA-MA sample. Similarly, the GMA standard displayed a clear spot under UV light, while no distinct spot was observed in the EPL-GMA sample at the corresponding position (Figure S3b). These results indicate that after thorough dialysis, residual MA and GMA monomers in both HA-MA and EPL-GMA were below the TLC detection limit (<0.1% *w*/*w*).

**Figure 1:**
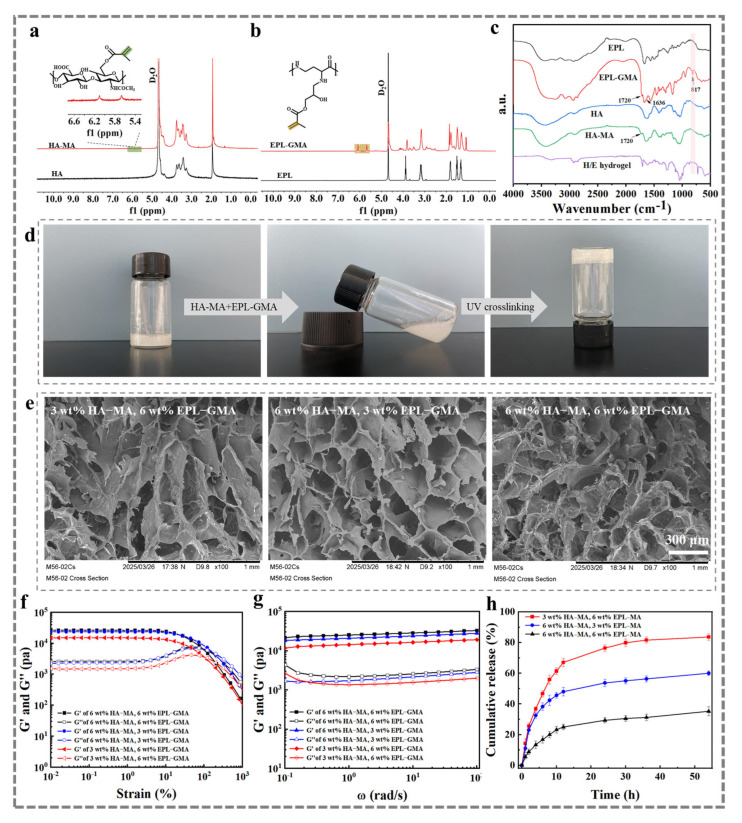
Hydrogel formation and characterization. ^1^H NMR spectra of HA-MA (**a**) and EPL-GMA (**b**). The green and yellow boxes mark the characteristic proton peaks of the carbon-carbon double bonds (C=C) introduced by methacrylation and grafting modification, respectively. (**c**) FTIR spectra of HA, HA-MA, EPL, EPL-GMA, and H/E hydrogel. (**d**) Gelation photos of the D@H/E hydrogel. (**e**) SEM images of D@H/E hydrogels with different formulations. (**f**,**g**) Strain and frequency scanning test of hydrogels with different formulations. (**h**) Cumulative release of DFO in hydrogels with different formulations at pH 7.4.

The structures of HA-MA and EPL-GMA were confirmed by ^1^H NMR and FTIR. As shown in Figure 1a, the signals that appeared at 3.20–3.90 ppm were assigned to the protons on the hexatomic ring of HA backbone; the peak at 1.93 ppm was assigned to the N-acetyl methyl protons of the HA backbone, while the weak peak at 1.87 ppm was assigned to the methacrylate methyl protons [38]; and the two distinctive peaks at 5.67 and 6.10 ppm were assigned to protons on the pendant vinyl groups [34], indicating the successful synthesis of HA-MA. The degree of substitution (DS) of HA-MA was calculated by comparing the integrated area of the methacrylate methyl protons (δ 1.87 ppm, 3H) to that of the N-acetyl methyl protons of the HA backbone (δ 1.93 ppm, 3H) [38]. The integration values were 1.00 for the methacrylate methyl signal and 8.24 for the HA methyl signal (Figure S4a), giving a DS of 12.1%. Figure 1b displays the ^1^H NMR spectra of EPL-GMA, the peak at 3.82 ppm was assigned to the proton of imide in the branch chain of EPL, the peak at 3.15 ppm was assigned to the ε-methylene protons of the lysine side chains, and the two distinctive peaks at 5.67 and 6.10 ppm were assigned to protons on the pendant vinyl groups, suggesting that the EPL has been modified by GMA [35]. The DS of EPL-GMA was calculated by comparing the integrated area of the vinyl protons (δ 5.67 ppm, 1H) to that of the ε-methylene protons of the lysine side chains (δ 3.15 ppm, 2H) [39]. The integration values were 1.00 for the vinyl signal and 11.97 for the ε-methylene signal (Figure S4b), giving a DS of 16.7%. Additionally, the FTIR spectra confirmed successful synthesis: HA-MA exhibited the characteristic ester C=O stretching band at 1720 cm^−1^, while EPL-GMA showed the ester C=O stretching band at 1720 cm^−1^ along with the C=C stretching of the pendant methacrylate groups at 1636 cm^−1^ and the C=C–H stretching band at 814 cm^−1^ [35].

### 3.2. Preparation and Characterization of H/E and D@H/E Hydrogels

Hydrogels can be formed by mixing the HA-MA and EPL-GMA solutions in equal volumes followed by irradiation under a 365 nm laser for 10 s (Figure 1d). FTIR spectroscopy was performed to confirm the structure of H/E hydrogel and the successful incorporation of DFO into the hydrogel network. As shown in Figure 1c, compared with EPL-GMA, the disappearance of the C=C–H stretching band at 814 cm^−1^ in the H/E hydrogel confirmed crosslinking of the double bonds. The FTIR spectra of D@H/E revealed distinct changes upon drug loading (Figure S5). Specifically, compared with the pristine H/E hydrogel, the D@H/E group exhibited increased intensities of the peaks at 1700 cm^−1^ (ν(C=O), amide I), 3327 cm^−1^ (ν(N–H) + ν(O–H)), 2932 cm^−1^ (ν_asym_(CH_2_)), 2861 cm^−1^ (ν_sym_(CH_2_)), and 1477 cm^−1^ (δ(C–H)) [40,41]. These enhancements indicate the successful and stable integration of DFO into the H/E hydrogel system. SEM images show that the hydrogels had a loose and porous network structure with a pore diameter of approximately ~200–350 μm (Figure 1e), which is conducive for the exchange of oxygen and nutrients when the hydrogels are used as wound dressings. For different formulations of the hydrogel, the pore size is also different. To further investigate the microstructural characteristics, quantitative analysis of the pore size and porosity was performed using ImageJ software based on the SEM images. The average pore diameter and porosity were measured as 347.8 ± 45.4 μm and 62.18 ± 1.75% for the H/E hydrogel with 3 wt% HA-MA and 6 wt% EPL-GMA; 240.4 ± 25.1 μm and 55.26 ± 1.64% for the hydrogel with 6 wt% HA-MA and 3 wt% EPL-GMA; and 193.8 ± 16.5 μm and 46.73 ± 1.19% for the hydrogel with 6 wt% HA-MA and 6 wt% EPL-GMA, respectively (Figures S6 and S7). This trend demonstrates that as the total polymer concentration increases, the hydrogel network becomes significantly denser with smaller pore sizes and lower porosity, which is primarily attributed to the increased cross-linking density within the matrix as higher concentrations of HA-MA and EPL-GMA provide more reactive functional groups per unit volume. From a biomedical perspective, these interconnected porous structures with pore sizes exceeding 100 μm are essential for diabetic wound dressings, as they effectively facilitate exudate absorption, maintain a moist healing environment, and provide sufficient channels for gaseous exchange and nutrient transport [42].

Rheological analysis revealed that the hydrogels maintained a high modulus within the strain range of 0.01–100%, whereas a significant decrease in modulus occurred beyond 100% strain (Figure 1f), indicating that the hydrogel network structure was disrupted under high strain conditions. The storage modulus (G′) at the plateau region was approximately 150 kPa, 250 kPa, and 260 kPa for hydrogels composed of 3 wt% HA-MA and 6 wt% EPL-GMA, 6 wt% HA-MA and 3 wt% EPL-GMA, and 6 wt% HA-MA and 6 wt% EPL-GMA, respectively. These values are comparable to those of Gelatin Methacryloyl (GelMA) hydrogels (15% wt% GM-60, 1.7 ± 0.15 × 10^4^ Pa), a widely used benchmark in bioapplications [43], indicating good elasticity of the hydrogels. Frequency sweep measurements over 0.1 to 100 rad s^−1^ showed that G′ consistently exceeded the loss modulus (G″) (Figure 1g), confirming the formation of a stable elastic network [44]. Moreover, G′ remained independent of angular frequency once the hydrogels were stably formed, demonstrating their robust structural integrity. This relatively high storage modulus provides a functional advantage for diabetic wound management, as it enhances the structural integrity of the dressing against mechanical deformation and external friction during clinical use. Furthermore, this modulus range aligns with the mechanical requirements for mimicking dermal tissue, ensuring that the hydrogel provides a stable and supportive microenvironment for wound repair.

The swelling and degradation properties of the hydrogels were analyzed. Hydrogels have excellent water absorption capacity. As shown in Figure S8, the water absorption swelling ratios of hydrogels can reach over 450% within 1 h. In a high-sugar environment, the swelling ratios of hydrogels differ slightly based on polymer concentration, and those with higher concentrations swell at a relatively slower rate. After 10 h, all hydrogels reach swelling equilibrium, and their water absorption swelling ratios reach over 620%. After the hydrogels reach swelling equilibrium, the degradation performance test is continued. As shown in Figure S9, the degradation behavior of hydrogels with different polymer concentrations are basically the same. The hydrogels demonstrated good stability with an over 90% of the retention mass after an incubation in PBS for 36 days. This may be due to the stability of the carbon-carbon single bonds formed by double bond crosslinking and their resistance to hydrolysis.

The H/E hydrogels also have good tissue adhesion. As shown in Figure S10, hydrogel formed in situ on porcine skin can firmly adhere to the surface of the porcine skin and will not fall off at various twisting angles. As shown in Figure S11, hydrogels with different formulations all exhibited good tissue adhesion strength, and higher total polymer concentrations further enhanced the adhesive performance. In addition, as shown in Figure S12, the hydrogels also demonstrated favorable compressive behavior, indicating good compressibility. These results collectively suggest that the hydrogels possess satisfactory mechanical properties.

### 3.3. Sustained Drug Release of Hydrogels

The loaded DFO was released from the hydrogel in a sustained manner, and the release rate decreased with increasing polymer concentration. As shown in Figure 1h, the cumulative release of DFO in D@H/E hydrogels containing 6 wt% HA-MA and 6 wt% EPL-GMA was 35.2 ± 2.9% at 54 h, which was much lower than that of the other two hydrogels with lower polymer concentrations. This might be due to the smaller pore size of the hydrogels with higher polymer concentrations, as shown in the SEM images. When the total polymer concentration kept constant, the cumulative release of DFO in D@H/E hydrogels containing 6 wt% HA-MA and 3 wt% EPL-GMA (60.0 ± 1.5%) was much lower than that in D@H/E hydrogels containing 3 wt% HA-MA and 6 wt% EPL-GMA (83.6 ± 2.1%), which may be due to the strong electrostatic interaction between HA-MA and DFO. The sustained drug release capability of the hydrogels enables them to continuously provide therapeutic effects during the wound repair process. Considering the comprehensive properties of the hydrogels, including pore size, porosity, elastic modulus, mechanical performance, and drug release behavior, the H/E hydrogel formulation composed of 6% HA-MA and 3% EPL-GMA was selected for subsequent cell and animal experiments.

### 3.4. Antioxidant Capability of Hydrogels

**Figure 2:**
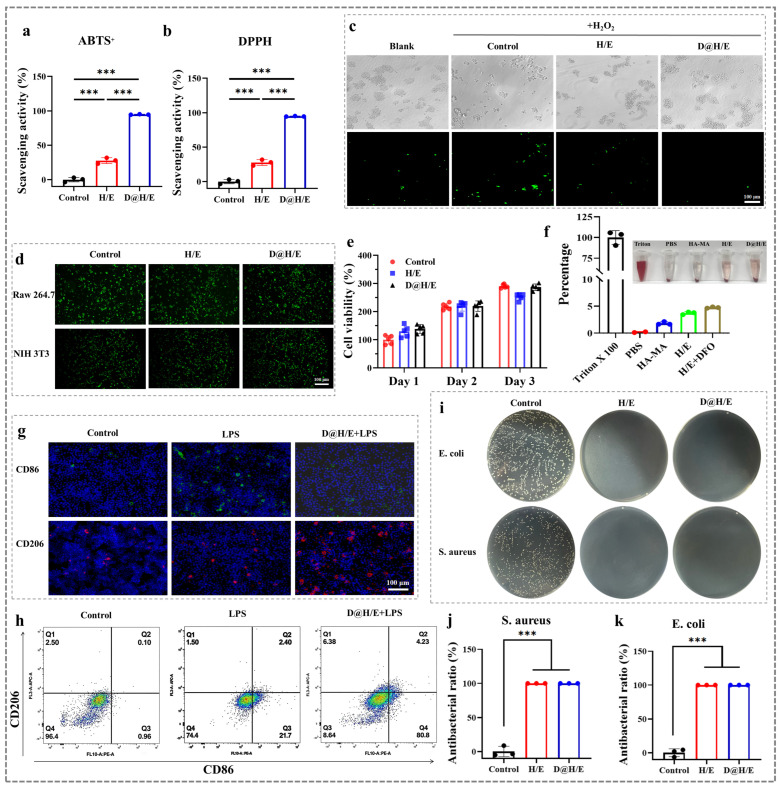
The hydrogels showed good antioxidant capability, good biocompatibility, ability to promote M2 polarization, and excellent antibacterial ability. (**a**,**b**) DPPH and ABTS^+^ free radical scavenging efficiency of the hydrogels. (**c**) Images of ROS signals in Raw 264.7 cells treated with H_2_O_2_ and subsequently treated with different hydrogels. (**d**) Live/dead staining of NIH 3T3 and Raw 264.7 cells incubated with hydrogel extracts. (**e**) CCK-8 assays of NIH 3T3 cells incubated with hydrogel extracts. (**f**) Hemocompatibility of the hydrogels. (**g**) Immunofluorescence staining of CD86 and CD206 for Raw 264.7 cells with different treatment. (**h**) Flow cytometry analysis of CD86 and CD206 staining for Raw 264.7 cells. (**i**–**k**) The antibacterial activity of the hydrogels against *S. aureus* and *E. coli. n* ≥ 3, *** *p* < 0.001.

High levels of ROS have been found in diabetic wounds, because hyperglycemia can affect the mitochondrial electrical transport chain and produce a large amount of ROS, thus leading to oxidative stress damage and further exacerbating inflammation and affecting angiogenesis [45]. Therefore, hydrogel dressings with good antioxidant properties play an important role in wound healing. The free radical scavenging ability of the hydrogels was evaluated through DPPH and ABTS^+^ free radical scavenging tests. As shown in Figure 2a and Figure 2b, the free radical scavenging abilities of H/E hydrogel for DPPH and ABTS^+^ were 21.9 ± 4.2 and 27.6 ± 4.2%, respectively; and the free radical scavenging abilities of D@H/E hydrogel for DPPH and ABTS^+^ were 80.8 ± 3.1% and 94.7 ± 0.5%, respectively. The free radical scavenging ability of D@H/E hydrogel is significantly higher than that of H/E hydrogel, which is attributed to the high antioxidant activity of DFO [46] and its inherent ability to remove free radicals through amino groups [47]. Furthermore, the ability of the hydrogels to protect cells from ROS damage was evaluated using DCFH-DA as the ROS probe. As shown in Figure 2c, compared with the control group, Raw 264.7 cells treated with H_2_O_2_ exhibited bright green fluorescence, indicating that the intracellular ROS level increased after H_2_O_2_ treatment. Fluorescence quenching and significant reduction in DCF fluorescence intensity were observed in cells treated with H/E and D@H/E hydrogels, suggesting that the hydrogels can effectively protect cells from oxidative stress damage. All these results indicate that the hydrogels exhibit excellent antioxidant ability.

### 3.5. Biocompatibility of Hydrogels

Good biocompatibility is a fundamental requirement for hydrogels as wound dressings. The biocompatibility of the hydrogels was evaluated through cytotoxicity and hemolysis tests. As shown in Figure 2d, after incubation for 24 h, both NIH 3T3 and Raw 264.7 cells treated by H/E and D@H/E hydrogels exhibited a large number of viable cells (green) and an extremely small amount of dead cells (red). In addition, the results of CCK-8 assay displayed that during the three days of co-incubation with the hydrogels, NIH 3T3 cells showed a good cell viability comparable to that of the control group (Figure 2e), suggesting that the hydrogels have good cytocompatibility. The hemocompatibility of the hydrogels was also evaluated. As shown in Figure 2f, the red blood cells treated by Triton X-100 showed a bright red color, indicating that the red blood cells were destroyed. The red blood cells treated by H/E and D@H/E hydrogels show a light pink color and the hemolysis ratio was less than 5%, indicating good hemocompatibility of the hydrogels. All these results showed that the hydrogels had excellent biocompatibility.

### 3.6. Hydrogels Promoted the Polarization of M2 Macrophage

Chronic inflammation is another characteristic of the microenvironment of diabetic wounds, which is also an important factor hindering the healing of diabetic wounds [4]. In the later stage of inflammation, pro-inflammatory macrophages (M1) transform into anti-inflammatory macrophages (M2), promoting the release of anti-inflammatory factors and the generation of ECM, preparing the wound for the stage of proliferation and remodeling [48]. However, in diabetic wounds, the transformation of macrophages from M1 to M2 phenotype is blocked, resulting in abnormal accumulation of inflammatory cells within the wound and amplification of the inflammatory response [49]. To investigate whether the hydrogel can promote the polarization of macrophages from M1 to M2 phenotype, the expression of CD86 (a marker of M1) and CD206 (a marker of M2) on the cell surface was detected by immunofluorescence staining under inflammatory conditions. As shown in Figure 2g, compared with the control group and the LPS group, only a small amount of CD86 (green) was expressed in the D@H/E hydrogel group, and the expression of CD206 (red) significantly increased. The results indicate that the D@H/E hydrogels can effectively promote the polarization of macrophages from M1 to M2 phenotype, which can help diabetic wounds pass through the inflammatory stage and accelerate the wound healing.

### 3.7. Antibacterial Activity of Hydrogels

The persistent hyperglycemia in diabetic wounds increases the risk of bacterial infection, hinders the resolution of the inflammatory response, and impedes wound healing [50]. Therefore, an ideal wound dressing should possess excellent antibacterial properties. The antibacterial performances of the hydrogels were evaluated using the agar plate culture method. As shown in Figure 2i–k, both H/E and D@H/E hydrogels exhibited excellent antibacterial activity against *S. aureus* and *E. coli*, with an antibacterial ratio of 99.9% and 99.8%, respectively. This is because the positively charged amino groups of EPL can interact with the negatively charged bacterial membranes through electrostatic forces, thus destroying the bacterial membrane, and killing the bacteria [51]. The results indicate that H/E and D@H/E hydrogels have outstanding antibacterial properties and can effectively treat bacterial infections in diabetic wounds, creating a favorable microenvironment to promote wound healing.

### 3.8. Hydrogels Promoted Cell Migration and Tube Formation

**Figure 3:**
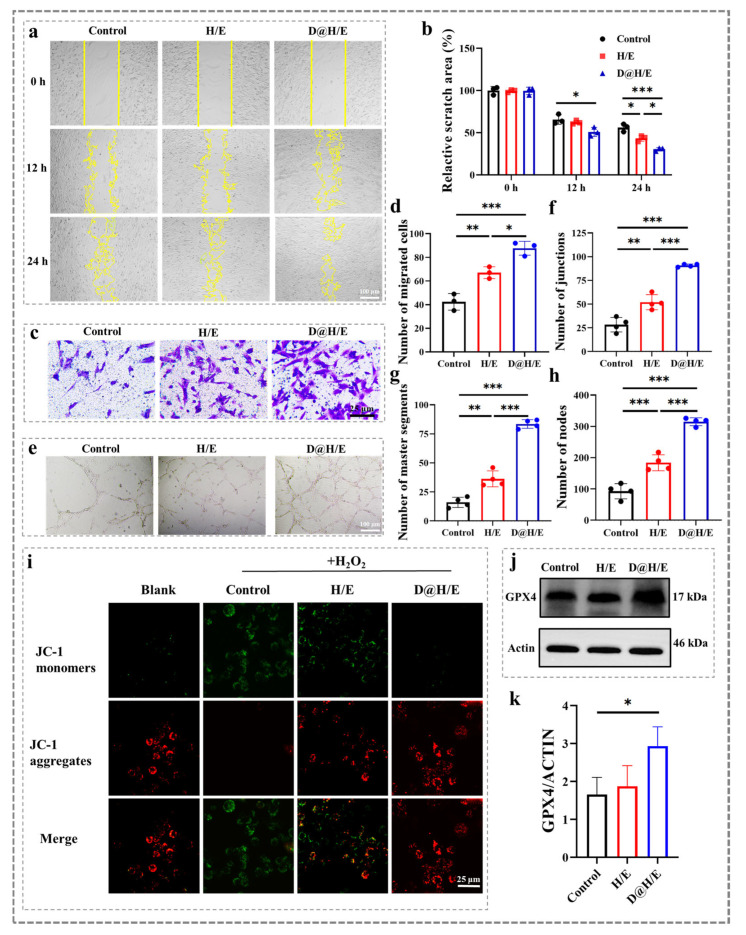
The hydrogels promote cell migration and tube formation, up-regulate the expression of GPX4 to alleviate ferroptosis and protect mitochondria from damage. (**a**) Representative images of NIH 3T3 migration treated by hydrogel extracts. (**b**) Statistical analysis of the relative scratch area of the cell scratch wounds. (**c**) Representative images of NIH 3T3 cells migrated to the lower chamber. (**d**) Statistical analysis of cell number migrated to the lower chamber. (**e**) Representative images of tube formation in HUVECs treated by hydrogel extracts. (**f**–**h**) Statistical analysis of the number of junctions, master segments, and nodes. (**i**) Images of JC-1 signals in Raw 264.7 cells treated by different hydrogels. (**j**) Representative blots of GPX4 in the regenerated tissues with different treatment. (**k**) The relative expression of GPX4 in each group. *n* ≥ 3, * *p* < 0.05, ** *p* < 0.01, *** *p* < 0.001.

Cell migration plays a crucial role in wound repair as it lays the foundation for the proliferation of fibrous tissues [52]. The effects of the hydrogels on cell migration were evaluated through cell scratch assay and Transwell assay. As shown in Figure 3a, the scratch area of all experimental groups gradually decreased with increasing time. The scratch area at 24 h was 30.4 ± 2.1% in the D@H/E group, which was significantly less than that in the H/E hydrogel group (43.3 ± 3.5%) and the control group (56.1 ± 4.7%) (Figure 3b). This was attributed to the ability of DFO to promote cell proliferation and migration [53,54]. In addition, compared with the control group, the number of migrated cells was significantly increased in H/E and D@H/E groups (Figure 3c,d).

Damage of the vascular system is one of the key factors hindering the diabetic wound repair [55]. The ability of the hydrogel to promote angiogenesis was evaluated by tube formation test using HUVECs, as the formation of endothelial cell tubes is crucial for angiogenesis during wound healing. As shown in Figure 3e–h, compared with the control group, the H/E and D@H/E hydrogel groups exhibited well-developed tubular networks, with significantly increased numbers of junctions, master segments and nodes. The D@H/E hydrogel group demonstrated the best tube formation ability, which was attributed to the angiogenesis effect of DFO.

### 3.9. Hydrogels Alleviated Ferroptosis

Studies have shown that diabetes-induced lipid peroxidation can promote ferroptosis, a new form of cell death related to β-cell dysfunction, insulin resistance, and diabetic cardiomyopathy [56,57]. Ferroptosis is dependent on the availability of ROS and iron, damages the functions of endothelial cells and fibroblasts, and hinders wound healing [58]. Therefore, reducing ferroptosis is beneficial to improve the healing of diabetic wounds. As an iron chelating agent, DFO in the hydrogel can effectively chelate iron ions and reduce the concentration of iron ions, thereby alleviating ferroptosis. Ferroptosis can cause mitochondrial damage, which can lead to the opening of the mitochondrial permeability transition pore, thus causing a decline in the mitochondrial membrane potential [59,60]. Therefore, the ability of hydrogels to alleviate ferropotosis was evaluated by the mitochondrial membrane potential test using JC-1 assay. Under normal conditions, the mitochondrial membrane potential is relatively high, and the JC-1 dye forms aggregates and emits red fluorescence. On the contrary, when the membrane potential is abnormally reduced, the dye forms monomers and emits green fluorescence, indicating a change in permeability in the mitochondrial membrane [61]. As shown in Figure 3i, in the H_2_O_2_ treated group, the green fluorescent signals were significantly increased, and the red fluorescent signals were significantly weakened or even disappeared, indicating mitochondrial damage in the oxidative stress environment. In contrast, the H/E group showed a slightly increased red fluorescent and decreased green fluorescent, indicating that the H/E hydrogels can alleviate mitochondrial damage at some extent. This may be due to the antioxidant property of the hydrogels. Furthermore, the DFO@H/E group showed the most significant increase in red fluorescence signal and even disappeared green fluorescence signal, indicating that DFO@H/E hydrogels can reduce the depolarization of mitochondrial membrane potential in oxidative stress environment, and maintain the polarization of mitochondrial membrane potential at a normal level. The capacity of DFO@H/E hydrogels to inhibit mitochondrial damage may be contributed to the antioxidant property of hydrogels as well as the iron chelation ability of DFO [32].

In addition, the amounts of GPX4 proteins in the regenerated tissues with different treatments were tested by Western blotting. GPX4 is a lipid hydroperoxidase that can scavenge excess lipid peroxides and is a critical regulator of ferroptosis [62,63]. In the hyperglycemia environment, the content and activity of GPX4 protein are significantly reduced in endothelial cells, resulting in an excessive accumulation of ROS and Fe^2+^, which are considered to be the key triggering factors for ferroptosis [64]. Western blotting analysis showed a much darker blot of GPX4 protein in the DFO@H/E group (Figure 3j), compared with the control group; and the expression of GPX4 protein was significantly up-regulated in the DFO@H/E hydrogel group (Figure 3k), further confirming that the DFO@H/E hydrogels can effectively alleviate ferroptosis in diabetic wounds, and thus promoting wound repair.

### 3.10. Hydrogels Enhanced the Diabetic Wound Healing in Mice

**Figure 4:**
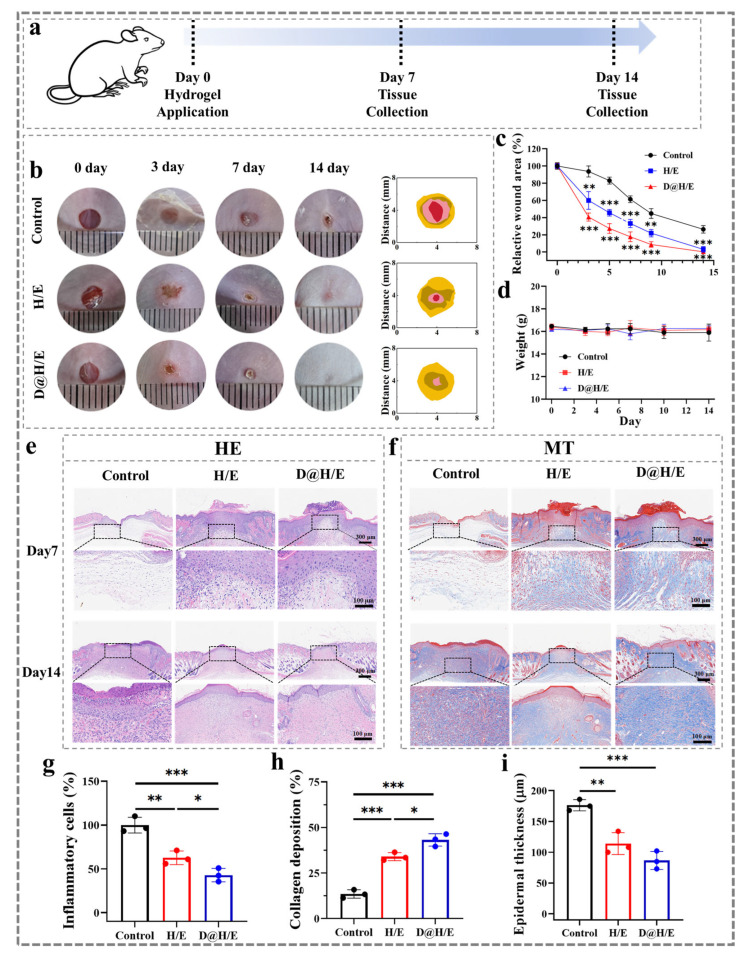
Hydrogels promoted wound healing in STZ-induced hyperglycemic mice. (**a**) Schematic illustration of the experiment procedures. (**b**) Representative photos of wound sites and the healing trajectory after different treatments. (**c**) Area of wounds in different groups over time, ** *p* < 0.01, *** *p* < 0.001 compared with control group. (**d**) Body weight of mice after different treatments over time. (**e**,**f**) Representative HE and MT images in different groups on day 7 and 14. Statistical analysis of inflammatory cells (**g**), collagen deposition (**h**), and epidermal thickness (**i**) on day 14. *n* = 3, * *p* < 0.05, ** *p* < 0.01, *** *p* < 0.001.

To evaluate the effectiveness of the hydrogel in diabetic wound healing, a STZ-induced diabetic wound model was established. A full-thickness skin wound (~4 mm in diameter) was created on the back of each BALB/c diabetic mouse. The hydrogel was cross-linked in situ at the wound site, and the repair effect of the hydrogel on the diabetic wound healing process was then observed. The experimental process was shown in Figure 4a. Figure 4b presented representative photos of the wounds at 0, 3, 7, and 14 days after different treatments, and the wound healing trajectory of each group is also analyzed. The results show that the wound closure rates in the H/E and D@H/E groups were significantly faster than those in the control group. Representatively, the relative wound areas on day 5 were 45.6 ± 3.8 and 27.3 ± 5.7% in the H/E and D@H/E group, respectively, which were significantly lower than that in the control group (83.0 ± 4.0%) (*p* < 0.01, Figure 4c). On day 7, the average wound area was 61.3 ± 3.9% in the 3M treated control group, which was significantly higher than those in the H/E and D@H/E groups (33.2 ± 4.8 and 17.7 ± 5.6%, respectively). On day 14, the wounds in the D@H/E group were almost completely closured, and only 3.1 ± 3.3% of unclosed wounds remained in H/E group, while the wound relative wound areas in the control group remained high as 26.3 ± 4.3%. In addition, during the treatment period, the body weight of the mice in all groups showed no significant change (Figure 4d), indicating that the hydrogels have good biocompatibility.

### 3.11. Histopathological Assessment of the Regenerated Tissues

The quality of the regenerated skin tissues in each group was further evaluated through histopathological analysis. Figure 4e and Figure 4f show the HE and MT staining images of the regenerated tissue at the 7th and 14th day, respectively. on the 7th day, the wounds in the H/E and D@H/E groups showed obvious granulation tissue and re-epithelialization compared with the control group. On the 14th day, obvious scabs and a large number of inflammatory cells could still be observed in the regenerated tissues of the control group, indicating that the wound was still in the inflammatory stage and had not yet progressed to the next stage. However, the number of inflammatory cells in the wounds of the H/E and D@H/E groups was significantly lower than that of the control group (Figure 4g), suggesting that the wounds had successfully passed the inflammatory stage and had basically completed re-epithelialization. Moreover, the thickness of the epithelium in the H/E and D@H/E groups was significantly lower than that of the control group (Figure 4e), indicating that the epithelium in the regenerated tissue was relatively mature [65]. MT staining showed that collagen deposition in the regenerative tissues could be observed in all groups. Compared with the control group, the collagen deposition in the H/E and D@H/E groups was significantly higher (Figure 4f), and the collagen arrangement was more regular and orderly. In addition, the D@H/E group also showed obvious new blood vessels and hair follicle formation, displaying the best repair effect among these groups.

### 3.12. Promoted M2 Macrophage Polarization of the Regenerated Tissues

**Figure 5:**
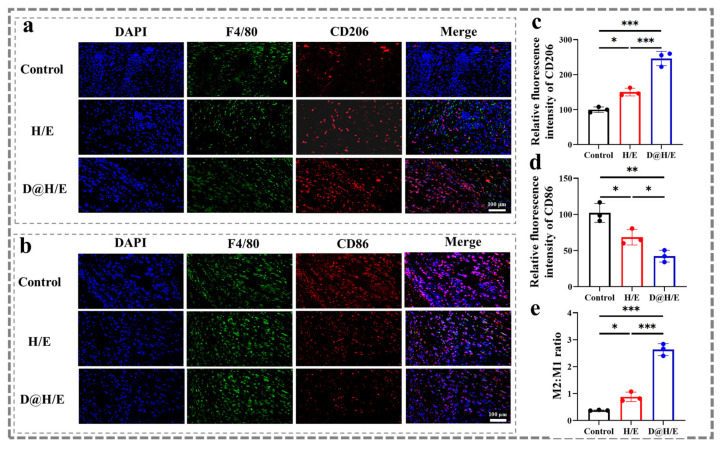
Hydrogels regulated the inflammatory response in vivo by promoting the polarization of M2 macrophages. (**a**) Immunofluorescence images of macrophages stained with F4/80 (green) and CD206 (red) in regenerated tissues on day 7. (**b**) Immunofluorescence images of macrophages stained with F4/80 (green) and CD86 (red) in regenerated tissues on day 7. Scale bar 100 μm. (**c**) Statistical analysis of M2 macrophage populations. (**d**) Statistical analysis of M1 macrophage populations. (**e**) The population ratio of M2/M1. *n* = 3, * *p* < 0.05, ** *p* < 0.01, *** *p* < 0.001.

To further evaluate the inflammatory response in the regenerated skin tissue, immunofluorescence staining of CD206 for M2 macrophages, CD86 for M1 macrophages, and F4/80 for panmacrophages was employed, since the prolonged inflammation is contributed to the blocked transformation of the M1 macrophage to M2 macrophage phenotype in the hyperglycemia environment [66]. As shown in Figure 5a–d, the expression level of M2 macrophages in the control group was relatively low with weak fluorescence of CD206, and the expression level of M1 macrophages was high with strong fluorescence of CD86. However, the expression levels of M2 macrophages were significantly high in the H/E and D@H/E groups, and the M1 macrophages were significantly low. These results indicated that the wounds of the control group were still in the inflammatory stage, while the wounds in the H/E and D@H/E groups had entered into the next healing stage, being consistent with the results of histomorphological analysis. This may be due to the antioxidant and anti-inflammatory properties of the hydrogels. In addition, the D@H/E group showed the highest expression levels of M2 macrophages and the population ratio of M2 to M1 macrophages (Figure 5e), which might be one of the reasons why it has the best wound healing effect among these groups.

### 3.13. Enhanced Neovascularization and Cell Proliferation of the Regenerated Tissues

**Figure 6:**
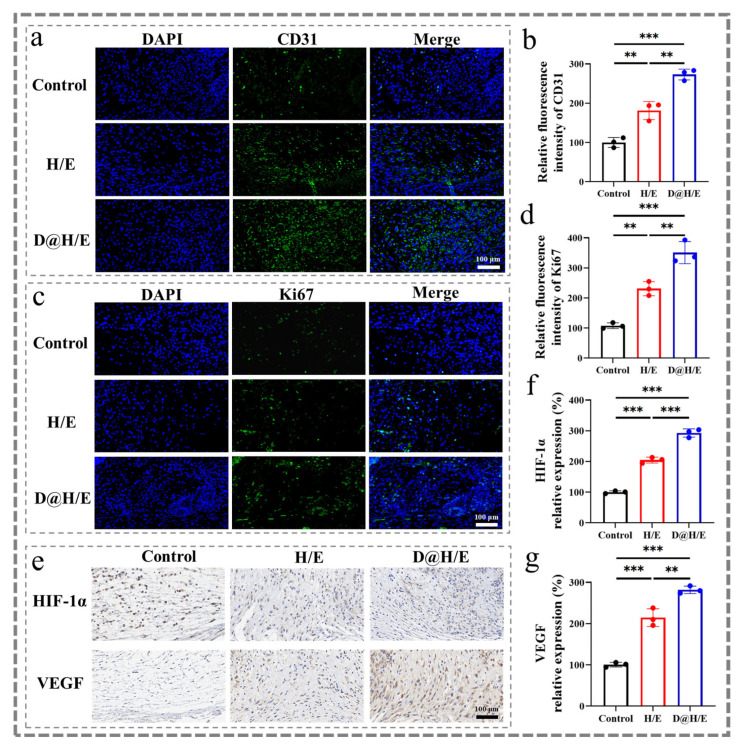
Hydrogels promote cell proliferation and angiogenesis in regenerated skin tissue by upregulating angiogenesis related factors. (**a**) Immunofluorescence images of CD31 in regenerated tissues on day 7. (**b**) Statistical analysis of CD31. (**c**) Immunostaining images of HIF-1α and VEGF in regenerative tissues on day 7. (**d**,**e**) Statistical analysis of HIF-1α and VEGF. (**f**) Immunofluorescence images of Ki67 in regenerated tissues on day 7. Scale bar 100 μm. (**g**) Statistical analysis of Ki67. *n* = 3, ** *p* < 0.01, *** *p* < 0.001.

Angiogenesis has a significant impact on the healing process as it can transport oxygen and nutrients to the injured tissues and promote their recovery [67]. The hyperglycemia environment can impair angiogenesis, thereby hindering the healing of diabetic wounds. Studies showed that M2 macrophages can produce anti-inflammatory and growth factors to reduce inflammation and promote angiogenesis [68]. The hydrogels can promote the polarization of M2 macrophage, and they should also be able to promote the formation of new blood vessels at the wound site. Therefore, immunofluorescence staining of CD31 (a vascular endothelial-specific marker) was performed to evaluation the angiogenesis in the wounds. CD31 is expressed during the early development of blood vessels, and its expression level can be used to assess the formation of new blood vessels [69]. As shown in Figure 6a,b, compared with the control group, the fluorescence intensity of CD31 in the H/E and D@H/E groups was significantly high, indicating that the hydrogel can promote the expression level of CD31. Additionally, the expression level of CD31 in the D@H/E group was significantly higher than that in the H/E group, which might be contributed to DFO. DFO is a vascular growth promoter, which has the ability to upregulate HIF-1α and other key downstream angiogenic factors such as VEGF [70]. Thus, immunohistochemical staining of HIF-1α and VEGF was further employed to evaluate the pro-angiogenic ability of hydrogels. As shown in Figure 6c–e, compared with the control group, the expression levels of HIF-1α and VEGF in the H/E and D@H/E groups were significantly up-regulated. The expression levels of HIF-1α and VEGF in the D@H/E group was the highest, which might be another of the reasons why it has the best wound healing effect among these groups.

Cell proliferation is necessary for the rapid closure of wounds [52]. Thus, immunofluorescence staining of Ki67 (a marker of cell proliferation) was performed to evaluation the cell proliferation in the wounds. The results displayed that the H/E and D@H/E hydrogel dressings could promote the proliferation of cells and accelerate wound repair. As shown in Figure 6f,g, the expression levels of Ki67 in the H/E and D@H/E groups were significantly up-regulated than that in the control group, indicating that the cell proliferation is good at the wounds treated with hydrogel dressings. The ability of hydrogels to promote cell proliferation may be due to the HA, which has been reported to improve the proliferation and migration of fibroblasts [71].

## 4. Conclusions

In this study, a multifunctional HA-MA/EPL-GMA hydrogel loaded with DFO was developed via rapid UV in situ crosslinking as an advanced dressing for diabetic wound repair. This system successfully addresses the multi-faceted pathological challenges of the diabetic wound microenvironment. It exhibits excellent biocompatibility, broad-spectrum antibacterial activity, and exceptional ROS scavenging capabilities. In vivo evaluations in STZ-induced diabetic mice confirmed that the hydrogel significantly accelerates wound closure by orchestrating multidimensional synergies: promoting M2 macrophage polarization to resolve persistent inflammation, upregulating GPX4 to alleviate ferroptosis, and enhancing the expression of relevant factors (HIF-1α, VEGF, CD31) to reconstruct functional vascular networks. Thus, this hydrogel provides a promising prospect for the treatment of chronic diabetic wounds.

## Data Availability

The datasets generated and/or analyzed during the current study are available from the corresponding authors upon reasonable request.

## Supplementary Materials

The following supporting information can be downloaded at: https://www.mdpi.com/article/10.3390/pharmaceutics18040473/s1, Figure S1: Standard curve of DFO detected using FeCl_3_; Figure S2. The schematic diagram of the synthesis route of HA-MA and EPL-GMA; Figure S3. TLC plate of MA, HA-MA extracts, GMA and EPL-GMA extracts; Figure S4. Expanded ^1^H NMR spectra of HA-MA and EPL-GMA showing the integrated area ratios of specified protons for DS calculation; Figure S5. FTIR spectra of DFO, H/E and D@H/E; Figure S6. Pore size of different hydrogel formulations; Figure S7. Porosity of different hydrogel formulations; Figure S8. The swelling properties of hydrogels with different formulations; Figure S9. The degradation properties of hydrogels with different formulations; Figure S10. Photographs of the D@H/E hydrogel adhered to porcine skin; Figure S11. Adhesion strength of hydrogel; Figure S12. Compression stress–strain curve of hydrogel.
