# Supplementary material for: A Multifunctional ε-Polylysine/Hyaluronic Acid Hydrogel Promotes Diabetic Wound Healing by Orchestrating Multidimensional Synergy
Source: Pharmaceutics. 2026 Apr 13;18(4):473. doi: 10.3390/pharmaceutics18040473 (PMC13119098; doi:10.3390/pharmaceutics18040473)
Supplement: Supplementary file 1 [file pharmaceutics-18-00473-s001.zip › pharmaceutics-4218411-supplementary.pdf]

Supplementary Materials: A Multifunctional ε-Polylysine/Hyaluronic Acid Hydrogel Promotes Diabetic Wound Healing by Orchestrating Multidimensional Synergy

Zelong Li [a], Yiqin Wang [a], Yifan Zhou [a], Hongze Liang [a], Xianwu Chen [b], Xiao Wang [c], Ziyu Liu [d*] and Lingling Zhao [a*]

[a]School of Materials Science and Chemical Engineering, Ningbo University, Ningbo, Zhejiang 315211, China

[b] The Affiliated Hospital of Medical School, Ningbo University, Ningbo 315211, China

[c] Health Science Center, Ningbo University, Ningbo, Zhejiang 315211, China.

[d] Beijing Advanced Innovation Center for Biomedical Engineering, School of Engineering Medicine, Beihang University, Beijing, 100191, China

Corresponding authors: Ziyu Liu (Email: liu_ziyu@buaa.edu.cn) and Lingling Zhao (email: zhaoling-ling@nbu.edu.cn)

Experimental

Swelling property test. The freeze-dried hydrogel was immersed in 10 mL of PBS (pH 7.4, 0.01 M) and incubated at 37°C. At each time point, the hydrogel was taken out and the excess water on the surface was gently removed by filter paper, and then weighed. The swelling ratio (SR) of the hydrogel is calculated using formula (1).

$$SR=\frac{W_t-W_0}{W_0}\times 100\% \qquad (1)$$

Where W_t represents the weight of the hydrogel at time *t*, and W_0 represents the initial weight of the freeze-dried hydrogel.

Degradation property test. The degradation behavior of the hydrogel is tested after the hydrogel reaches the swelling equilibrium. In brief, the hydrogel was immersed in 10 mL of PBS (pH 7.4, 0.01 M) and incubated at 37°C. At each time point, the hydrogel was taken out and the excess water on the surface was gently removed by filter paper, and then weighed. The degradation rate (DR) of the hydrogel is calculated using formula (2):

$$DR=\frac{W_s-W_d}{W_s}\times 100\% \qquad (2)$$

Where W_s represents the weight of the hydrogel at swelling equilibrium, and W_d represents the weight of the hydrogel at a specific time of degradation.

Tissue Adhesion and Mechanical Performance test. The EPL-GMA and HA-MA solutions were applied onto the porcine skin. In situ photopolymerization was carried out using ultraviolet light. Then, the adhesive properties of the hydrogels were macroscopic qualitative evaluated by a visceral adhesion test in porcine skin adhesion under torsion stress and joint adhesion. Adhesive strength was quantified via lap-shear tests, in which two pieces of porcine skin were bonded with the hydrogel and pulled apart on the testing machine. Compressive me-

chanical tests were performed using a SUNS electronic universal testing machine. Square samples (10 mm side length) were compressed to 70% strain at 10 mm min^{-1}.

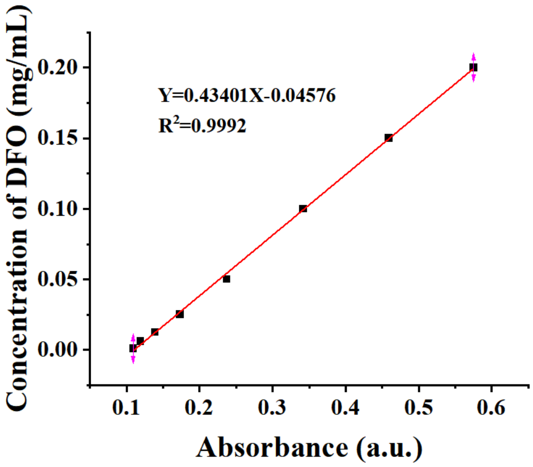

Figure S1. Standard curve of DFO detected using $FeCl_3$.

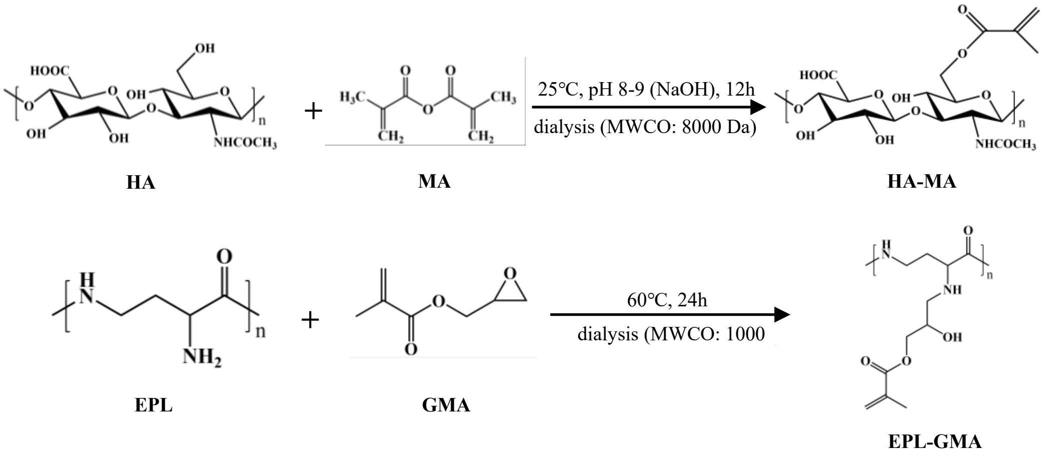

Figure S2. The schematic diagram of the synthesis route of HA-MA and EPL-GMA.

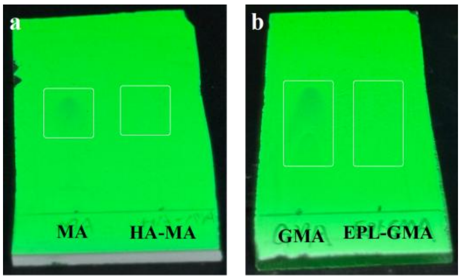

Figure S3. (a) TLC plate of MA and HA-MA extracts, MA standard (clear spot) vs. HA-MA extract (no spot). (b) TLC plate of GMA and EPL-GMA extracts, GMA standard (clear spot) vs. EPL-GMA extract (no spot).

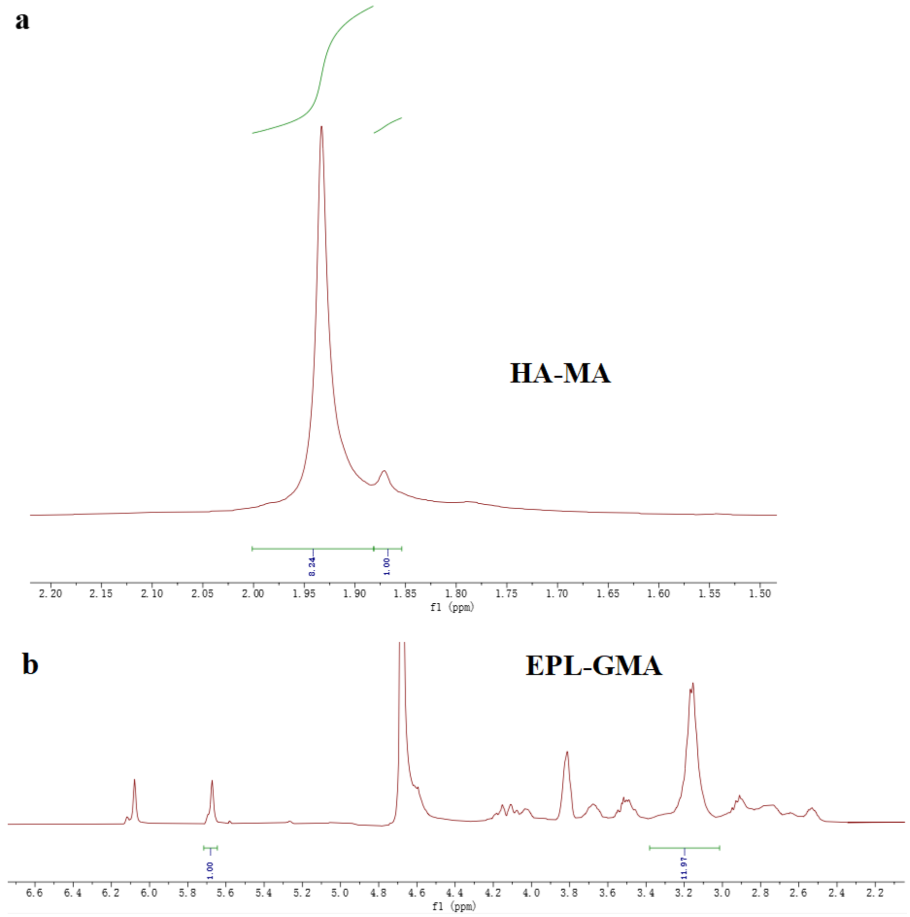

Figure S4. Expanded ^{1}H NMR spectra of HA-MA (a) and EPL-GMA (b) showing the integrated area ratios of specified protons for DS calculation.

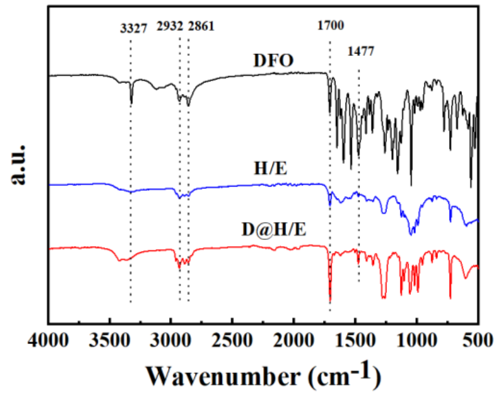

Figure S5. FTIR spectra of DFO, H/E and D@H/E.

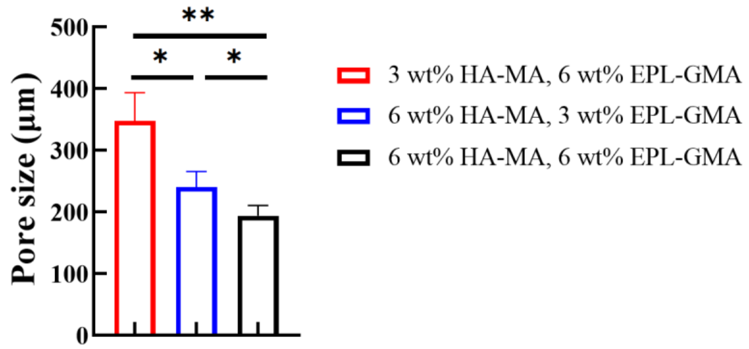

Figure S6. Pore size of different hydrogel formulations.

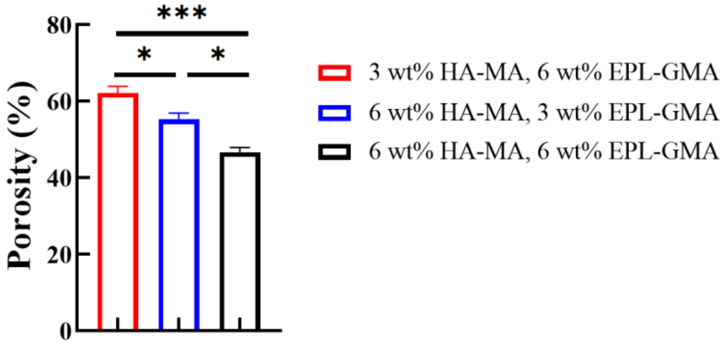

Figure S7. Porosity of different hydrogel formulations.

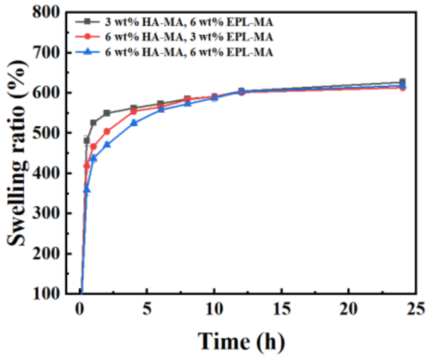

Figure S8. The swelling properties of hydrogels with different formulations.

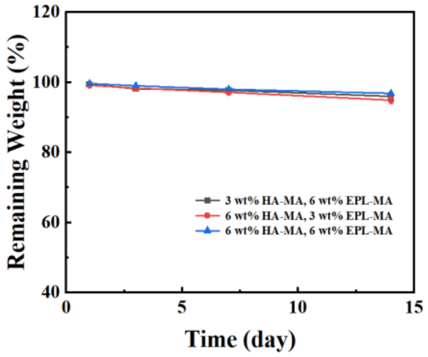

Figure S9. The degradation properties of hydrogels with different formulations.

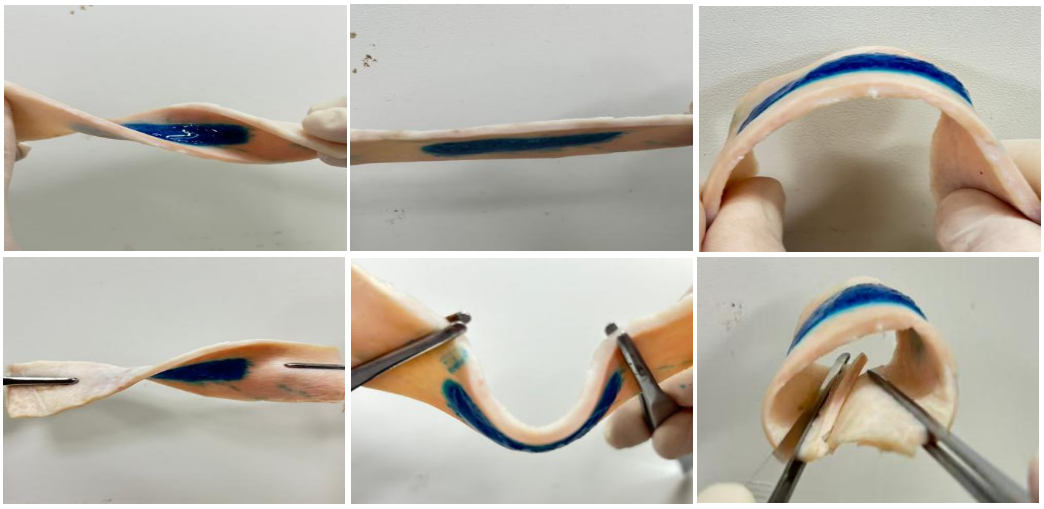

Figure S10. Photographs of the D@H/E hydrogel adhered to porcine skin.

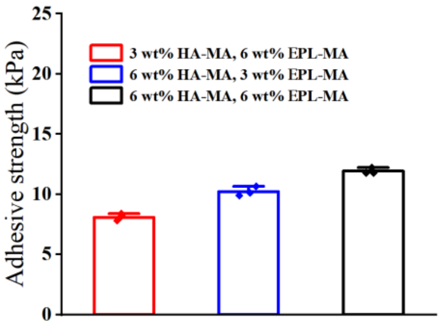

Figure S11. Adhesion strength of hydrogel.

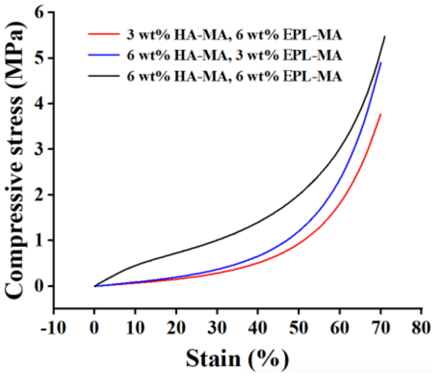

Figure S12. Compression stress-strain curve of hydrogel.
